# Transcriptome analysis of life stages of the house cricket, *Acheta domesticus*, to improve insect crop production

**DOI:** 10.1038/s41598-020-59087-z

**Published:** 2020-02-26

**Authors:** Brenda Oppert, Lindsey C. Perkin, Marcé Lorenzen, Aaron T. Dossey

**Affiliations:** 1 USDA ARS Center for Grain and Animal Health Research, 1515 College Ave, Manhattan, KS 66502 USA; 2 Department of Entomology and Plant Pathology, North Carolina State University, Raleigh, NC 27695 USA; 3 All Things Bugs LLC, 755 Research Parkway, Suite 465, Oklahoma City, OK 73104 USA

**Keywords:** Biotechnology, Genetics, Molecular biology

## Abstract

To develop genetic resources for the improvement of insects as food, we sequenced transcripts from embryos, one-day hatchlings, three nymphal stages, and male and female adults of the house cricket, *Acheta domesticus*. A draft transcriptome was assembled from more than 138 million sequences combined from all life stages and sexes. The draft transcriptome assembly contained 45,866 contigs, and more than half were similar to sequences at NCBI (e value < e^−3^). The highest sequence identity was found in sequences from the termites *Cryptotermes secundus* and *Zootermopsis nevadensis*. Sequences with identity to *Gregarina niphandrodes* suggest that these crickets carry the parasite. Among all life stages, there were 5,042 genes with differential expression between life stages (significant at p < 0.05). An enrichment analysis of gene ontology terms from each life stage or sex highlighted genes that were important to biological processes in cricket development. We further characterized genes that may be important in future studies of genetically modified crickets for improved food production, including those involved in RNA interference, and those encoding prolixicin and hexamerins. The data represent an important first step in our efforts to provide genetically improved crickets for human consumption and livestock feed.

## Introduction

The human population is expanding along with increasing world hunger driven by climate change and political conflict, amid substantial levels of biodiversity loss and mass extinction of species worldwide^1–4^. Consumption is driving the increased need for energy, land, and water. Animal livestock requires approximately 70% of the land devoted to agriculture and uses 70% of fresh water^5,6^. Agricultural pollution due to methane emissions from animals has been significantly underestimated, as increases in emissions over a recent ten-year period were correlated to an increase in the number of traditional farm animals^7^. Clearly, expanding livestock production to meet all the needs of the growing human population will have considerable costs and negative environmental impacts. Thus, it is important to identify sources of protein that produce lower levels of pollution and lessen destruction of habitat and natural resources.

Insects offer a sustainable solution as an alternative food source, requiring 10–50% less water and land per pound of protein compared to other animals, with higher growth and reproductive rates^8^. For example, food input to weight for cattle is approximately 7:1, 4:1 for pork, 2:1 for poultry, and less than 2:1 for fish^9^. By comparison, crickets convert approximately 1.25:1 feed to body mass. Insects also contain vital nutrients, including the eight essential amino acids, vitamin B12, riboflavin, vitamin A and minerals^10,11^. Thus, mass-produced farm-raised insects hold great promise for use as ingredients rich in essential nutrients for food products.

Crickets in general, and in particular field crickets from the *Gryllus* spp, are a model for orthopteran studies as well as insect development and limb regeneration^12^. Genetic editing of *G. bimaculatus* has been performed using TALENs and zinc-finger nucleases^13^, as well as CRISPR/Cas-based approaches^14^. RNA interference (RNAi) has been successful in *G. bimaculatus*^15,16^, and transgenic *G. bimaculatus* have been produced using eGFP-marked *piggyBac* elements^17^. Similar approaches for the house cricket, *Acheta domesticus*, also have been successful in our laboratories (unpublished data).

*A. domesticus* is one of the most widely farmed insects, particularly in North America and Europe. Farmed crickets likely originated in Asia, but now constitute a thriving pet/reptile feeder insect market worldwide. Crickets like *A. domesticus* are high in protein (about 70% by dry weight), hemimetabolous (having only egg, nymphal and adult stages with no larvae or pupae), have a short life cycle (around 5 wks), are prolific (females lay more than 1,500 eggs), and are the basis for an emerging and vibrant insect-based food industry^18^. However, as with other modern approaches to livestock management, genetic tools are needed to improve insects as food crops. For example, genetic modifications could provide disease resistance while improving the protein content of crickets.

The only transcriptome study for *A. domesticus* to date is of the head and thorax^19^, but there are transcriptome data from other cricket species^20–34^ (Table 1). Robust genetic engineering will require detailed genomic and transcriptomic data. In particular, life stage-specific expression patterns of various genes/promoters/regulatory elements within the species will be needed to determine the timing and levels of expression for potential gene targets. These data can be used to mitigate cricket mortality due to pathogens, increase nutritional value, increase growth rate and overall productivity, and optimize the timing of production and harvest. Developing the tools for genetic engineering in insects provides an open-ended opportunity to use insects for food, feed and other valuable applications.

**Table 1:** Publications of transcriptome studies in cricket species.

Genus species	Common Name	Tissues	Goal	Reference
*Acheta domesticus*	House cricket	Head and thorax	Centromere evolution	^19^
*Apteronemobius asahinai*	Mangrove cricket	Female heads	Circadian rhythm	^20^
*Gampsocleis gratiosa*	Chinese bush cricket	Adult whole body	Microsatellites	^21^
*Gryllus bimaculatus*	Two-spotted cricket	Blastema	Leg regeneration	^22^
	Field cricket	Ovary and embryo	Database	^23^
		Prothoracic ganglion	Cell signaling	^24^
*Gryllus firmus*	Sand field cricket	Fat body, flight muscles	Wing polymorphism	^25^
*Gryllus firmus, G. pennsylvanicus*	Sand field cricket, Fall field cricket	Male accessory gland	Species evolution	^26^
		Hindgut & malpighian tubules	Cold-acclimation & database	^27^
*Gryllus rubens*	Southeastern field cricket	Life stages (eggs, 1-6 instar nymphs, adult male and female)	Database	^28^
*Gryllus veletis*	Spring field cricket	Male nymph fat body	Freeze tolerance	^29^
*Laupala eukolea*	Hawaiian swordtail cricket	Male and female juveniles and adults	Song evolution	^30^
*Teleogryllus commodus*	Australian black field cricket	Male and female brain	Developmental plasticity	^31^
*Teleogryllus emma*	Emma field cricket	Whole-body adult	Database & antimicrobial peptides against *E. coli*	^32^
*Teleogryllus oceanicus*	Oceanic field cricket	Testis, accessory gland, male adult remaining tissue	Species evolution	^33^
		Developing wing buds	Phenotype evolution	^34^

To address these goals, we analyzed the *A. domesticus* transcriptome at six time points throughout development: embryo; 1 d hatchlings; 1, 2, and 4 wk nymphs; and adult males and females. We identified genes that were highly expressed in each life stage for future work, in which promoters will be needed to drive expression of engineered transgenes. Gene expression was compared between developmental stages and male and female adults, and a few gene groups of interest were highlighted. This research lays the foundation for future research in cricket genetic transformation to improve nutritional value for human and animal consumption.

## Methods

### Tissue extraction and sequencing

Tissues were obtained from different life stages of cricket (embryos, 1 d hatchlings, 1, 2, and 4 wk nymphs, and male and female adults). Nymphs and adults were obtained from a cricket farm and shipped to the Center for Grain and Animal Health Research, (CGAHR), Manhattan, KS and North Carolina State University (NCSU). Embryos were collected from the offspring of adults. Four biological replicates for each life stage (except n = 3 for embryos and n = 2 for hatchlings) were flash frozen in liquid N_2_ and were stored at −80 °C. Total RNA was extracted from all samples using Tri-reagent and a Direct-zol kit (Zymo Research, Irvine, CA USA). Libraries were constructed from total RNA, barcoded, and quantitated on a NeoPrep (Illumina, San Diego, CA USA) using a NeoPrep library kit and standard protocols. In brief, the NeoPrep isolates mRNA via robotics, requiring 25–100 ng of total RNA per sample, and automates barcoding of libraries and normalization. Due to the lack of ribosomal RNA depletion kits for most insects, rRNA was not removed prior to library construction. Barcoded libraries were pooled and sequenced on a MiSeq (Illumina, 2 × 300 paired-end), with two technical replicates for each biological replicate. Sequencing metrics indicated that the total number of reads ranged from about 9 million for 1 d hatchlings to 25 million for 1 wk nymphs (Table 2A). Reads were submitted to NCBI under Bioproject PRJNA485997 (SRA and Biosample accession numbers are in Table 2A).

**Table 2:** Sequencing and assembly metrics.

A. Sequencing metrics for *A. domesticus* transcriptome sequencing of life stages (sum of all reads and total number of bases for each sample, N = biological replicates).					
Sample	N	Total Reads	Total (Mb)	NCBI SRA Accession #	Biosample Accession #
Embryo	3	16,938,084	7,176	SRR7692602	SAMN09832431
1 d Hatchling	2	8,986,264	3,272	SRR7692603	SAMN09832434
1 wk Nymph	4	25,334,406	9,749	SRR7692605	SAMN09832435
2 wk Nymph	4	24,052,818	10,187	SRR7692600	SAMN09832429
4 wk Nymph	4	20,285,520	8,690	SRR7692601	SAMN09832432
Female Adult	4	20,345,084	9,729	SRR7692604	SAMN09832433
Male Adult	4	22,281,716	9,481	SRR7692599	SAMN09832430
TOTAL	25	138,223,892	58,284		
**B. Assembly metrics for** ***A. domesticus*** **life stages transcriptome sequencing**.					
**Statistic**				**Number**	
Assembled reads				73,916,712	
Unassembled reads				32,619,909	
Reads excluded by sampling (removed)				31,541,615	
Adaptor (removed)				1,501	
Total				138,079,737	
# Contigs				45,866	
# Contigs > 1kb				26,665	

(**A**) Metrics for *A. domesticus* transcriptome sequencing of life stages (sum of all reads and total number of bases for each sample, N = biological replicates), and (**B**) Assembly statistics of the number of assembled and unassembled reads and the number of contigs obtained from the assembly of *A. domesticus* transcriptome life stage sequences.

### Bioinformatics

#### Assemblies

All sequence reads from *A. domesticus* life stages were assembled by SeqManNGen v.16 (DNAStar, Madison, WI USA) using the *De Novo* Assembly option on a MacPro with 128 GB RAM (Tables 2B, S1 Table). Approximately half of the total reads were assembled, and unassembled reads likely were in part due the heterogeneity of the genome. Reads removed during sampling occurred because the algorithm clusters similar reads of up to 100,000 reads, and thus reads after 100,000 were removed due to this limit. This Transcriptome Shotgun Assembly project has been deposited at DDBJ/EMBP/GenBank under the accession GHUU00000000 (SUB6156302). The version described in the paper is the first version, GHUU01000000. All contigs from the assembly were compared to NCBI databases (both Invertebrate Ref Seq and NR) using default E-value of e^−3^ in BLASTx^35^ and were mapped and annotated in OmicsBox^36^ v.1.1 (BioBam, Valencia, Spain). Contigs that were annotated as *Gregarina niphandrodes* were removed from the *A. domesticus* transcriptome assembly and submitted to TSA under the accession GHVX00000000 (SUB6289302). The version described in this paper is the first version, GHVX01000000.

To further analyze contigs from a draft transcriptome assembly that were annotated as *G. niphandrodes*, all sequence reads from *A. domesticus* life stages also were mapped to the *G. niphandrodes* genome sequence (accession GCA_000223845.4 GNI3), using the SeqManNGen Reference Guided Assembly option. There were 553,102 reads that mapped to 184/469 scaffolds in the *G. niphandrodes* reference assembly.

#### Gene expression analyses

Gene expression in each life stage was analyzed by ArrayStar (DNAStar). Reads from each developmental stage were aligned to the draft transcriptome and were normalized by RPKM^37^. Genes were annotated in ArrayStar by importing the OmicsBox annotation file. Differential gene expression across all developmental stages was evaluated by ANOVA, and significant differences were limited by a p < 0.05 threshold. Expression data of gene groups were visualized via bar graphs and Venn diagrams (Heirarchial clustering using Euclidean distance metric) within ArrayStar to highlight important differences in gene expression between life stages and sexes. We also extracted gene groups of interest and compared expression across life stages and sexes via heat map analysis in ArrayStar.

#### Gene annotation

Gene ontology (GO) enrichment analyses were performed in OmicsBox using the Fisher’s exact test enrichment analysis. For the first analysis, all genes with RPKM ≥ 1 for each developmental stage or sex were submitted as the test set and were compared to the reference set of all genes, using default values (FDR = 0.05). The enrichment analysis evaluated GO IDs from all GO categories (Biological Process, BP; Cellular Component, CC; Molecular Function, MF). Results were reduced to most specific terms (FDR = 0.05) and were visualized as a word cloud, with the size of the word reflecting the sequence count for each GO term relative to the counts of other words. The color of each word was generated randomly. Assignment of enzyme codes and KEGG pathway analysis (Kyoto Encyclopedia of Genes and Genomes^38^ licensed to USDA ARS) were conducted within OmicsBox.

### Ethical procedures

All animal handling and molecular biology procedures were approved by the KSU Institutional Biosafety Committee (IBC permit 1191).

## Results

**Figure 1:**
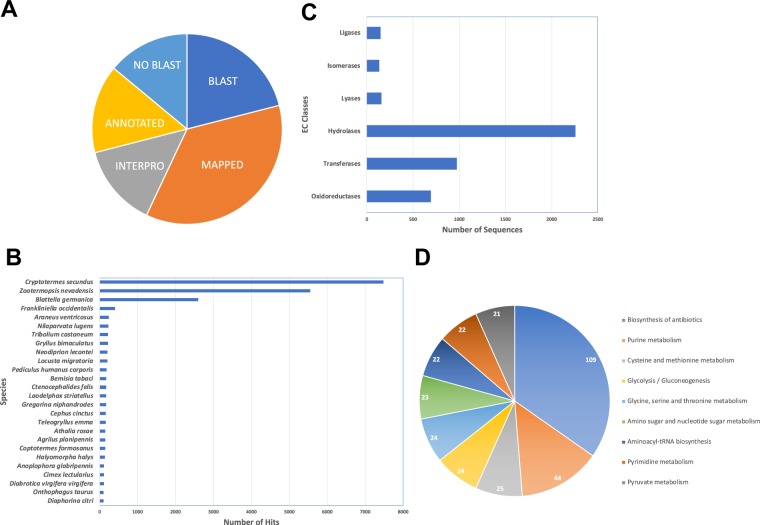
Annotation of contigs from the *A. domesticus* transcriptome assembly, obtained from transcript sequences from different life stages. (**A**) Distribution of sequences with BLAST hits, annotation, and GO mapping; (**B**) Top hits by species from the BLAST analysis of contigs from the *A. domesticus* transcriptome; (**C**) Distribution of enzyme codes in the *A. domesticus* transcriptome; (**D**) Top metabolic pathways supported by enzymes in the *A. domesticus* (pathways containing > 20 enzymes).

Transcripts were sequenced from developmental stages of *A. domesticus*, consisting of more than 138 million reads from embryos, 1 d hatchlings, 1-, 2-, and 4-wk nymphs, and male and female adults (Table 2A). Of these, approximately 74 million reads were assembled into a draft transcriptome, resulting in 45,866 contigs, with more than half greater than 1 kb (Table 2B). Contigs were submitted to OmicsBox for BLASTx analysis, Gene Ontology (GO) mapping, and annotation (Fig. 1A). More than half of the contigs (27,294) had a BLAST hit to databases, and 67% were annotated.

BLAST top hits included insects from orders Araneae, Blattodea, Coleoptera, Hemiptera, Hymenoptera, Isoptera, Orthoptera, Phthiraptera, Siphonaptera, and Thysanoptera (Fig. 1B). Almost half of the top hits were from termites (*Cryptotermes secundus* and *Zootermopsis nevadensis*). Only a small number of sequences (573) had identity to orthopteran species, *Gryllus bimaculatus, Locusta migratoria*, and *Teleogryllus emma*, and the former and latter were the only cricket species in the dataset. Interestingly, a subset of contigs (182) had hits to *G. niphandrodes*, suggesting that these crickets may have the associated gregarine parasite (S2 Table). The *G. niphandrodes* contigs were removed from the *A. domesticus* transcriptome and were analyzed separately. Overall, the data reflected the limited amount of genetic information available for cricket species in publicly available databases.

Mapping contigs from the *A. domesticus* transcriptome to enzyme codes (EC) identified sequences from EC classes hydrolases (2,257), transferases (970), oxidoreductases (692), lyases (154), ligases (148), and isomerases (131) (Fig. 1C). Enzymes from the dataset mapped to 128 metabolic pathways, as determined by KEGG pathway analysis (S3 Table). Purine metabolism was supported by the highest number of contigs (1,086). Remarkably, 109 of the *A. domesticus* enzymes mapped to the “Biosynthesis of Antibiotics” pathway (Fig. 1D). Other major pathways were: metabolism of purine and pyrimidine, cysteine, methionine, glycine, serine, and threonine, pyruvate, as well as amino and nucleotide sugars; glycolysis and gluconeogenesis; and aminoacyl-tRNA biosynthesis.

### Analysis of gene expression

**Figure 2:**
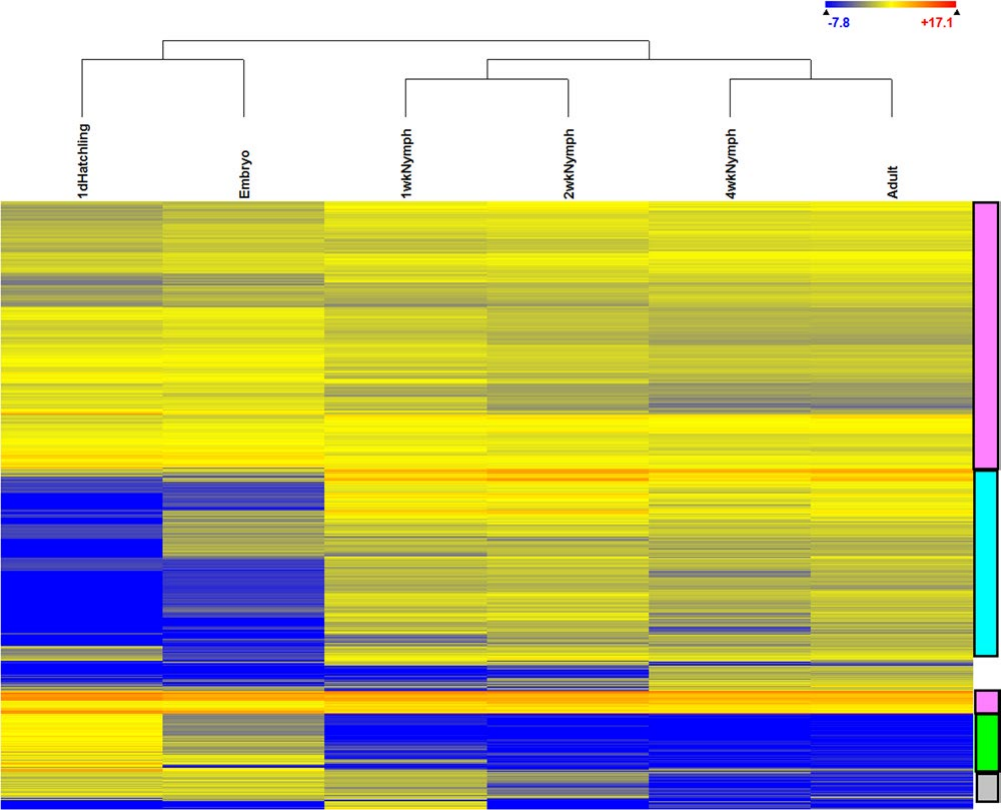
Differential expression of genes among life stages of *A. domesticus* (ANOVA, p < 0.01), with grouping of life stages above, and expression legend in upper right. Patterns of expression discussed in the text are in boxes to the right: moderate to high levels, pink; low levels in embryos and 1 d hatchlings but moderate to high in other life stages, turquoise; moderate to high levels in embryos and 1 d hatchlings but low levels in other life stages, green; and moderate expression in early stages (embryo, 1 d hatchling, and 1 wk nymph), grey. Identification of the contigs in this heat map are in S4 Table.

A comparison of the expression levels of genes that were significantly (p < 0.05) different among all life stages of *A. domesticus* was visualized in a heat map (Fig. 2, S4 Table). The data consisted of 5,042 genes, and expression patterns of embryo and hatchlings clustered into one group, whereas nymphs and adults clustered into another group. Overall, three patterns of expression emerged in the heat map: genes that were similarly expressed at moderate to high levels throughout all life stages (Fig. 2, legend on right, pink); genes that were expressed at low levels or not at all in embryos and hatchlings, but moderate to higher levels in other life stages (turquoise); and genes that were moderately expressed in embryos and 1d hatchlings, but low to no expression in other life stages (green). There was a small cluster of genes in 1 wk nymphs with expression more closely aligned with embryos and 1 d hatchlings than with the other life stages (grey). A large number of contigs in this group (2,114) had no blast hits (S2 Table). Many of the genes were ribosomal, housekeeping, or encoded structural components.

Genes that had high expression (RPKM > 5,000) in all developmental stages were identified as they may have promoters that will be useful in future work to develop transgenic strains (Table 3). One contig had a BLAST hit to a hypothetical protein (cl_605230_1) and was the most highly expressed in all life stages, and highest in hatchlings. Contigs also annotated as hypothetical proteins included cl_292231_9, highly expressed only in hatchlings, and cl_94434_1, highly expressed only in adults. Others contigs included those encoding actin (cl_890041_1), highly expressed in embryos, hatchlings, and 1 wk nymphs; superoxide dismutase (cl_378021_4), highly expressed in hatchlings and 1 wk nymphs; cytochrome c oxidase subunits I (cl_956902_2), highly expressed in nymphs and adults, and II (cl_283644_1), highly expressed in 1 and 2 wk nymphs and female adults; and cytochrome b (cl_108298_2), highly expressed only in 2 wk nymphs. Contigs cl_378021_3, cl_772328_1, and cl_378021_15 were highly expressed in embryos/1 wk nymphs, hatchlings, and hatchlings/4 wk nymphs/female adults, respectively, but they had no BLAST hits. The greatest number of highly expressed contigs (seven) were found in hatchlings.

**Table 3:** Highly expressed contigs (RPKM > 5,000) in life stages of *A. domesticus*.

Life Stage	Contig	Sequence Description	RPKM
Embryo	cl_605230_1	hypothetical protein	72,000
	cl_378021_3	---NA---	17,210
	cl_890041_1	Actin, muscle	6,178
1 D hatchling	cl_605230_1	hypothetical protein	136,500
	cl_890041_1	Actin, muscle	14,060
	cl_378021_15	---NA---	9,954
	cl_772328_1	---NA---	7,098
	cl_378021_4	superoxide dismutase	6,211
	cl_292231_9	hypothetical protein ALC62_00582	5,387
	cl_846226_4	Actin, muscle	5,134
1 wk nymph	cl_605230_1	hypothetical protein	82,120
	cl_956902_2	cytochrome c oxidase subunit I (mitochondrion)	8,949
	cl_283644_1	cytochrome c oxidase subunit II (mitochondrion)	7,846
	cl_378021_4	superoxide dismutase	6,762
	cl_378021_3	---NA---	6,385
	cl_890041_1	Actin, muscle	5,389
2 wk nymph	cl_605230_1	hypothetical protein	66,180
	cl_956902_2	cytochrome c oxidase subunit I (mitochondrion)	9,746
	cl_283644_1	cytochrome c oxidase subunit II (mitochondrion)	8,670
	cl_108298_2	AF248682_1cytochrome b (mitochondrion)	5,057
4 wk nymph	cl_605230_1	hypothetical protein	51,540
	cl_956902_2	cytochrome c oxidase subunit I (mitochondrion)	6,663
	cl_378021_15	---NA---	6,545
Adult female	cl_605230_1	hypothetical protein	59,340
	cl_956902_2	cytochrome c oxidase subunit I (mitochondrion)	7,785
	cl_378021_15	---NA---	6,107
	cl_94434_1	hypothetical protein X777_10552	6,044
	cl_283644_1	cytochrome c oxidase subunit II (mitochondrion)	5,553
Adult male	cl_605230_1	hypothetical protein	39,140
	cl_94434_1	hypothetical protein X777_10552	10,180
	cl_956902_2	cytochrome c oxidase subunit I (mitochondrion)	5,691

### Enrichment analyses

**Figure 3:**
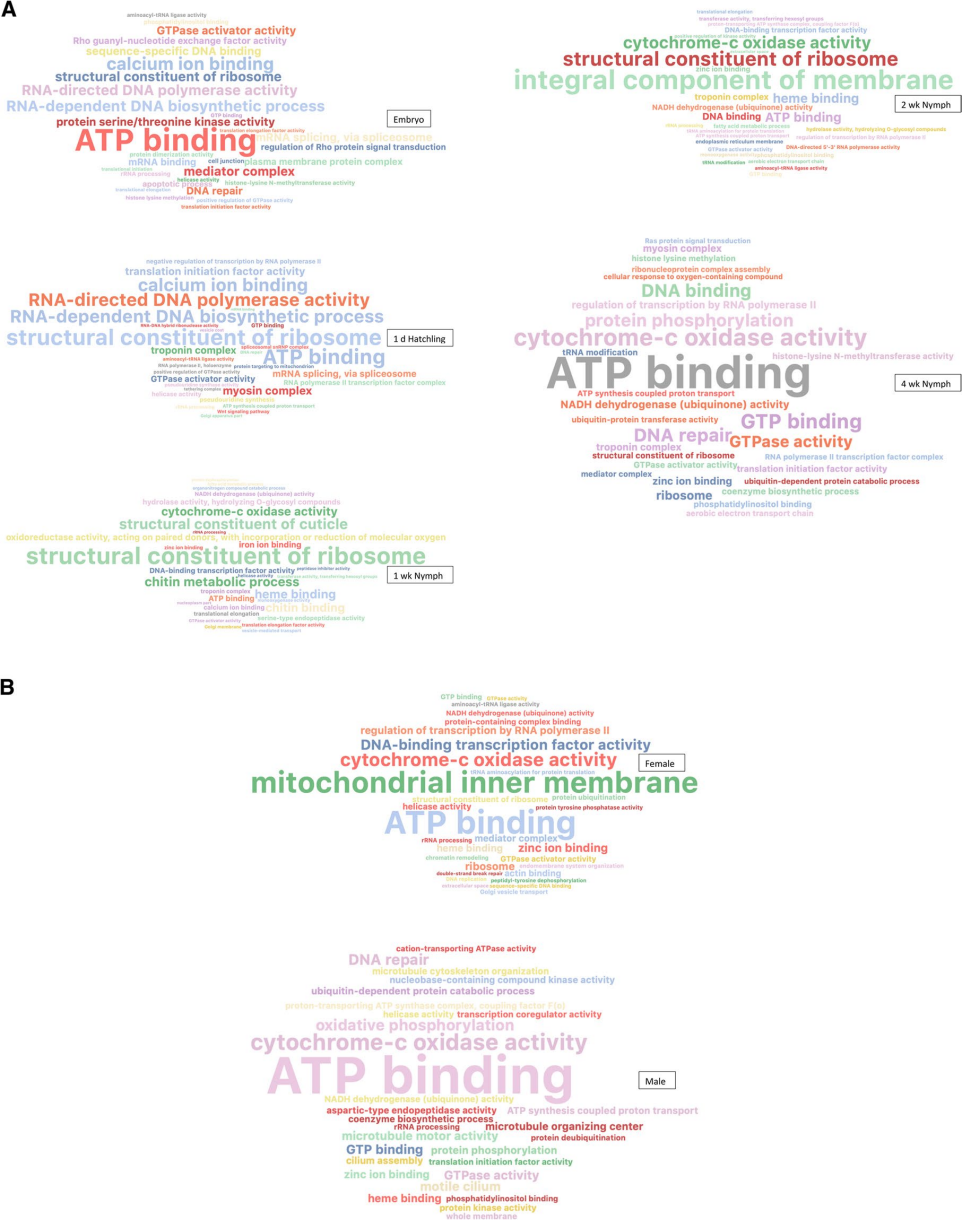
Enrichment of GO terms in different life stages or sexes of adult *A. domesticus*, as represented by word clouds. (**A)** Enriched GO terms, in embryo, 1 d hatchling, and 1, 2, 4 wk nymphs; (**B)**. Enriched GO terms in male and female adults. After filtering for RPKM ≥ 1, total number of genes in each set were: embryo, 30,899; 1 d hatchling, 29,113; 1 wk nymph, 31,557; 2 wk nymph, 29,924; 4 wk nymph, 27,842; female adult, 30,264; male adult, 31,441.

We also used an enrichment analysis of all *A. domesticus* genes filtered to RPKM $\ge \,$1 in each life stage to gain discrete snapshots into important functions via word clouds of GO terms (Fig. 3). The comparison of GO terms in embryos through 4 wk nymphs illustrated that early stages (embryos and hatchlings) were mostly inducing energy and biosynthetic processes, with terms like “ATP binding” and those associated with DNA polymerase, “calcium ion binding”, and “structural constituent of ribosome” more prevalent in 1 d hatchlings (Fig. 3A). In 1 wk nymphs, “structural constituent of ribosome” was most prominent, but chitin-related terms (“structural constituent of cuticle”, “chitin metabolic process”, chitin binding) are now emphasized, and to a lesser extent “heme binding” and “cytochrome-c oxidase activity”. All terms except chitin-related appear in 2 wk nymphs, but “integral component of membrane” was the most enriched term. In the last nymphal stage sampled (4 wk), the most important term was “ATP binding” and “cytochrome-c oxidase activity” that are indicative of energy production in the maturing cricket, and “GTP binding” and “GTPase activity” that suggest the importance of signaling processes.

Enriched GO terms also were compared in male and female *A. domesticus* adults (Fig. 3B). Males and females shared the highly enriched GO terms “ATP binding” and “cytochrome-c oxidase activity”. Interestingly, “mitochondrial inner membrane” was the most important term in females, but “DNA-binding transcription factor activity” and “regulation of transcription by RNA polymerase II” also were important. Processes associated with sperm formation in males may be reflected in the enriched terms “microtubule organizing center”, “microtubule motor activity”, “motile cilium”, and “cilium assembly”. These datasets highlight the dynamic nature of the transcriptome, changing dramatically across developmental stages.

### Prolixicin gene expression

**Figure 4:**
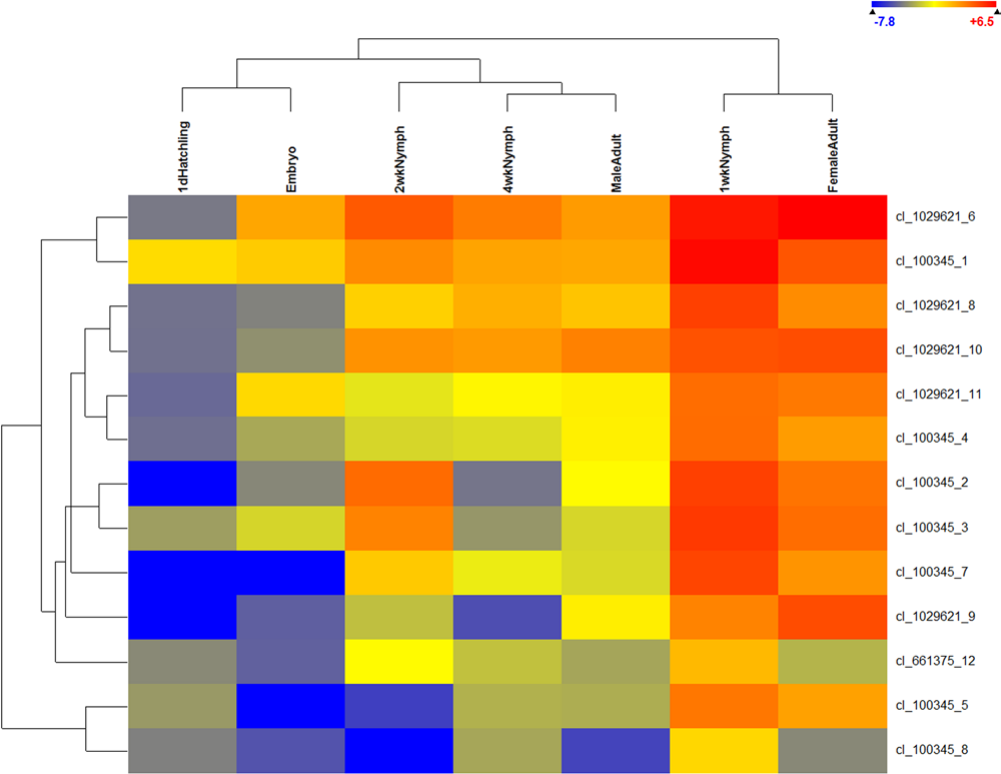
Heat map of prolixicin gene expression in different life stages or male and female adults of *A. domesticus*, with life stage grouping above, contig grouping on the left, expression legend upper right, and contig identification to the right.

Thirteen of the sequences in the antibiotic biosynthesis pathway from the KEGG analysis encoded the antimicrobial peptide prolixicin. Overall, expression of the prolixicin contigs was low to very low in embryos and 1 d hatchlings, respectively, but their expression ramped up dramatically in 1 wk nymphs, the earliest feeding stage that we analyzed (Fig. 4). In later stages (2 and 4 wk), prolixicin gene expression was more moderate. In adults, however, the expression of prolixicin genes in female was more similar to that of 1 wk nymph, whereas male expression was more similar to that of other nymphal stages. The exception was contig cl_100345_1, which was expressed at moderate to high levels in all developmental stages.

### Genes associated with RNA interference

A survey of contigs encoding proteins associated with RNA interference (RNAi) that are typically found in other organisms indicated that *A. domesticus* should have a robust RNAi response (Table 4). *A. domesticus* contigs were similar to argonaute-1 and 2, Dicer, PIWI, and RISC-loading complex. The expression patterns of RNAi contigs varied among the *A. domesticus* life stages, likely due to many of these being partial sequences and/or representing different isoforms. There was only one contig similar to argonaute-1 (cl_292728_11) with an e-value of 6.90^−107^ (*Z. nevadensis*) that represented a full-length transcript. Eleven transcripts were annotated as argonaute-2, four from the contig group cl_292728, three from cl_146309, and two from cl_405821. Dicer was represented by seven sequences, six from cl_230105. PIWI annotations were assigned to 11 sequences, three each from cl_173437 and cl_486080. There were six contigs with RISC-loading complex annotations, four from cl_348946.

**Table 4:** Sequences encoding typical RNAi genes in the *A. domesticus* transcriptome and relative expression in different life stages and sexes.

				RPKM (log_2_)						
						Nymph			Adult	
**Contig**	**Identification**	**E-Value**	**Top Hit**	**Embryo**	**1 d**	**1 wk**	**2 wk**	**4 wk**	**Female**	**Male**
cl_292728_11	Argonaute-1	6.90^-107^	*Zootermopsis nevadensis*	0.40	0.06	0.04	0.01	0.04	0.07	0.21
cl_146308_4	argonaute-2	9.70^-33^	*Ceratina calcarata*	0.01	0.08	0.06	0.02	0.01	0.02	0.01
cl_292728_12		9.80^-98^	*Sogatella furcifera*	2.69	1.04	0.05	0.20	0.26	0.07	0.21
cl_292728_13		3.60^-69^	*Cryptotermes secundus*	2.91	1.31	0.39	1.05	1.19	2.26	1.21
cl_615557_4		1.20^-31^	*Cryptotermes secundus*	1.25	0.01	0.24	0.28	0.22	1.42	1.38
cl_292728_1		0	*Locusta migratoria*	25.6	12.8	21.3	18.8	14.4	24.2	13.2
cl_146308_8		4.70^-60^	*Cryptotermes secundus*	0.01	0.01	0.02	0.02	0.01	0.04	0.01
cl_405821_2		1.30^-128^	*Zootermopsis nevadensis*	0.01	0.01	0.01	0.01	0.30	0.01	8.07
cl_405821_1		0	*Zootermopsis nevadensis*	0.05	0.03	0.01	0.01	1.00	0.01	30.6
cl_615557_1		0	*Zootermopsis nevadensis*	16.6	10.4	20.4	24.0	16.1	33.6	18.9
cl_146308_1		6.00^-161^	*Locusta migratoria*	20.3	15.3	51.4	30.2	15.9	34.5	20.6
cl_292728_5		1.30^-13^	*Nilaparvata lugens*	0.01	0.01	0.01	0.02	0.01	0.02	0.02
cl_230105_3	Dicer	2.30^-29^	*Dufourea novaeangliae*	0.01	0.01	0.01	0.01	0.22	0.01	11.6
cl_230105_2		8.70^-38^	*Neodiprion lecontei*	0.01	0.01	0.01	0.01	0.12	0.01	3.69
cl_230105_1		1.30^-72^	*Dufourea novaeangliae*	0.01	0.04	0.01	0.01	0.34	0.01	24.0
cl_255905_15		8.20^-15^	*Fopius arisanus*	2.43	2.14	0.26	0.01	3.47	0.96	4.44
cl_255905_5		1.10^-161^	*Cryptotermes secundus*	4.02	1.34	4.87	5.16	5.66	7.26	7.30
cl_255905_12		1.00^-119^	*Zootermopsis nevadensis*	2.56	3.06	3.93	3.99	5.35	7.19	5.34
cl_255905_10		1.10^-108^	*Cryptotermes secundus*	3.61	1.02	5.77	6.83	9.55	12.2	11.6
cl_149574_1		2.60^-111^	*Blattella germanica*	5.09	4.34	9.55	10.0	14.9	18.8	14.9
cl_65654_1	Piwi	1.00^-37^	*Gregarina niphandrodes*	0.01	0.01	2.02	0.16	0.07	0.56	0.02
cl_486080_12		8.40^-50^	*Cryptotermes secundus*	0.66	0.74	0.04	1.04	2.17	0.67	0.20
cl_486080_9		6.60^-59^	*Ostrinia furnacalis*	1.75	0.52	1.47	2.82	0.47	0.35	0.06
cl_486080_3		0	*Cryptotermes secundus*	4.58	2.02	3.45	3.74	6.50	11.1	5.51
cl_173437_1		0	*Gryllus bimaculatus*	0.01	0.03	0.08	1.71	0.36	23.1	0.01
cl_79968_1		5.30^-08^	*Anoplophora glabripennis*	0.03	0.01	2.70	0.48	0.13	0.02	3.13
cl_259332_2		3.00^-152^	*Gryllus bimaculatus*	0.10	0.05	2.02	1.90	0.15	0.03	6.01
cl_259332_1		0	*Gampsocleis gratiosa*	0.13	0.05	3.69	1.51	0.19	0.01	7.55
cl_173437_2		0	*Gampsocleis gratiosa*	0.42	0.08	3.48	0.05	0.51	0.03	6.44
cl_173437_3		3.90^-139^	*Gampsocleis gratiosa*	0.92	0.01	2.38	0.04	0.15	0.01	5.37
cl_275146_1		0	*Gryllus bimaculatus*	24.7	13.3	23.1	26.7	15.7	34.3	10.2
cl_178763_1	RISC-loading complex	3.90^-13^	*Saccoglossus kowalevskii*	0.02	0.01	0.01	0.04	1.21	0.01	49.27
cl_348946_30		2.90^-29^	*Cryptotermes secundus*	0.01	3.51	2.03	0.82	0.44	0.21	0.47
cl_348946_31		5.10^-29^	*Cryptotermes secundus*	0.16	1.64	1.82	0.36	2.63	3.63	0.90
cl_348946_11		2.70^-27^	*Cryptotermes secundus*	3.32	2.88	4.73	6.09	4.27	10.2	6.63
cl_348946_14		1.40^-169^	*Cryptotermes secundus*	11.6	8.35	12.3	13.4	7.85	18.9	8.23
cl_284228_1		2.40^-38^	*Zootermopsis nevadensis*	0.05	0.15	0.62	2.50	5.02	0.75	6.81

Multiple sequence alignments of the RNAi-associated contigs did not provide additional clarity (data not shown), and it is unclear if these represent alternative splicing and/or partial transcripts. However, based on expression patterns, three argonaute-2 contigs (cl_292728_1, cl_615557_1, and cl_146308_1) had high expression in all life stages and may represent isoforms (Table 4). Higher expression of Dicer, PIWI, and RISC-loading complex contigs were in cl_255905_10, cl_275146_1, and cl_284228_1, respectively, but BLAST analysis suggested that only the PIWI contig represents a full-length transcript.

### Genes encoding hexamerin 1

**Figure 5:**
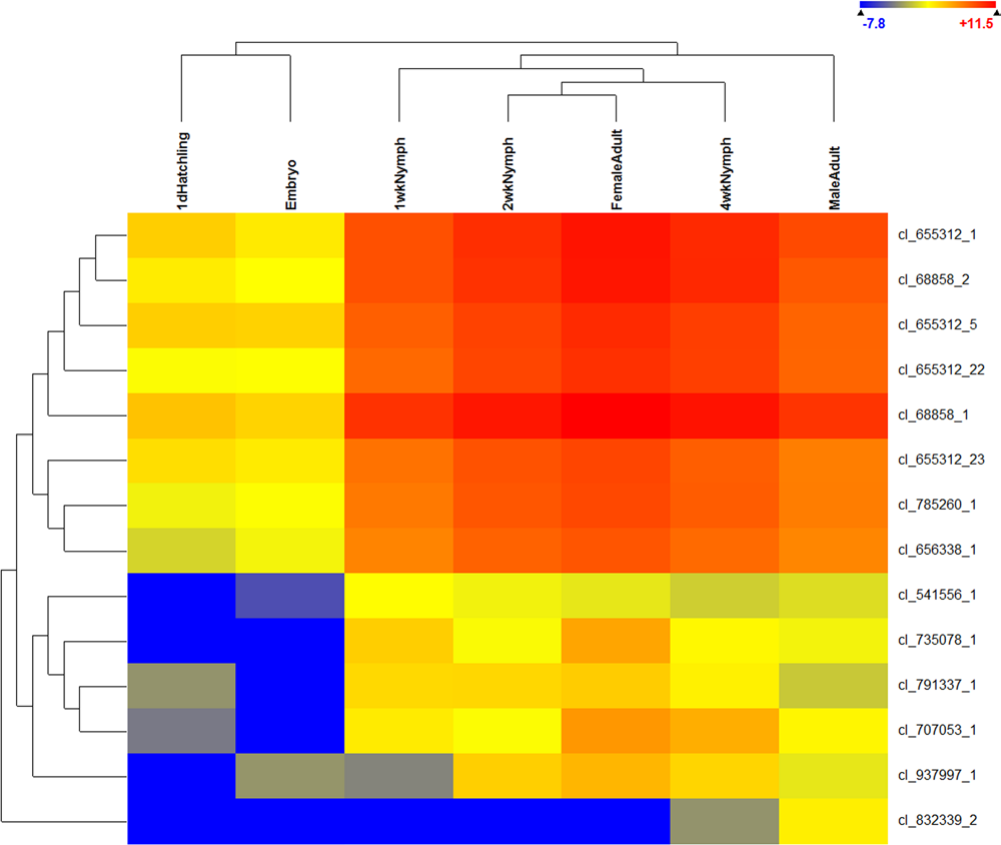
Heat map of hexamerin gene expression in different life stages or male and female adults of *A. domesticus*, with life stage grouping above, contig grouping on the left, expression legend upper right, and contig identification to the right. Identification of the contigs in this heat map are in S5 Table.

There were 101 contigs from the *A. domesticus* transcriptome annotated as a specific group of storage proteins, hexamerin, with hits to 13 different species (S5 Table). Expression of 14 hexamerin contigs were significantly different among life stages and sexes (ANOVA, p < 0.05), and a heat map depicting expression levels of these 14 contigs revealed two expression patterns (Fig. 5). The upper group was moderately to highly expressed in all life stages, whereas the lower group was expressed mostly in nymphs and adults, with the bottom sequence possibly exhibiting male-specific expression. Expression patterns of these hexamerin sequences were similar in embryos and hatchlings, whereas the expression patterns of nymphs and adults were similar.

### Genes from gregarines

**Figure 6:**
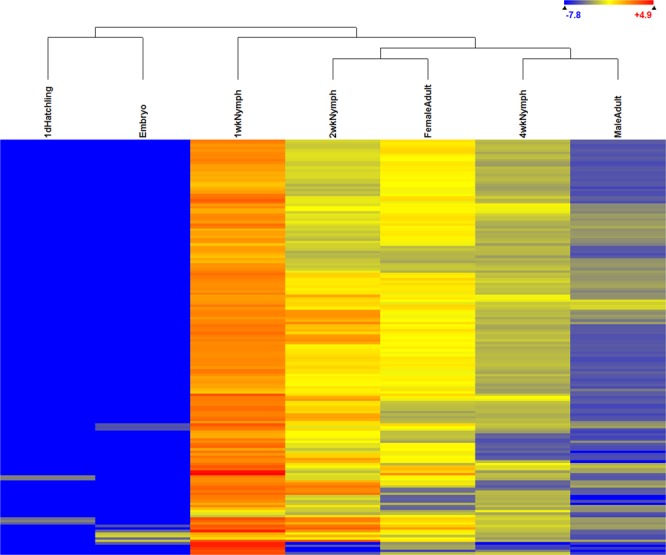
Heat map of the expression levels from *G. niphandrodes* contigs found *A. domesticus* life stages or male and female adults, with life stage grouping above and expression legend upper right. Identification of the contigs in this heat map are in S2 Table.

As mentioned above, a subset of contigs (0.04%) were annotated as transcripts from *G. niphandrodes* (S2 Table). Therefore, we performed a reference guided assembly of all reads extracted from the *A. domesticus* transcriptome to the *G. niphandrodes* genome assembly and identified about 0.04% of the reads that mapped to the gregarine reference genome (data not shown). These reads mapped to 39% of the genome scaffolds and suggest that these crickets carried the gregarine parasite. Examination of expression levels from these contigs in the cricket life stages and sexes indicated that there was low to no expression in the embryo and hatchlings, very high expression in the 1 wk nymph, and moderate expression levels in 2- and 4-week nymphs and female adults (Fig. 6). Expression level of *G. niphandrodes* contigs in male adults was lower than in nymphal stages or female adults.

## Discussion

In this study, we described a draft transcriptome from various life stages of *A. domesticus* and an additional set of contigs from *G. niphandrodes*. The data revealed a need for additional sequence data from other orthopterans, as the majority of hits from a BLAST of *A. domesticus* contigs came from two termite species, both with sequenced genomes^39,40^. Our transcriptome data identified potential genes in *A. domesticus*, and also important gene expression data among different developmental stages, as well as among male and female adult crickets. Patterns of expression indicated that embryos and 1 d hatchlings often clustered in expression analyses, whereas nymphs and adults usually had similar patterns. In addition, the transcriptome sequences are providing valuable information in assembly of the very large (approximately 2 Gb) and heterozygous *A. domesticus* genome (unpublished data).

Highly expressed genes (RPKM > 5,000) were found in all life stages and sexes, but more were found in hatchlings. One contig (cl_605230_1) was expressed much higher than all other contigs in all developmental stages, but the function of this gene is unknown, as it was annotated as a hypothetical protein in other insects. The *α−tubulin* promoter is frequently harnessed to drive transgene expression when creating transgenic insects^41^, but this gene was not found in our highly expressed dataset. Genes encoding other hypothetical proteins were highly expressed in hatchlings or adults and highlight the need to explore the functions of these genes in crickets and other insects. Superoxide dismutase, expressed at higher levels in hatchling and 1 wk nymphs (contig cl_378021_4), is an antioxidant enzyme in insects, and increased expression in early stages may be reflective of the onset of feeding. Increased expression of mitochondrial enzymes (cytochrome b and cytochrome c oxidase) in nymphs and adults reflect increased respiration in the later stages.

In looking at snapshots of gene expression at different life stages via word clouds, we discovered that processes in embryos and hatchlings were mostly associated with energy and biosynthetic production. Chitin-related terms appeared in the first nymphal stage, and terms in later stages demonstrated the relative increase of terms associated with structural ribosomes, membrane components, energy and respiration, and signaling. In female adults, ontology terms indicated enhanced processes in the mitochondrial inner membrane and transcription/translation. Energy and respiration functions also were enhanced in males, but we also found expected ontology terms related to sperm formation.

Our goal in this study was to obtain life stage specific expression patterns and annotate sequences encoding proteins that could be vital to the improvement of *A. domesticus* for food production, and also those that may be manipulated in the design of transgenic crickets. While there was a long list of candidate genes and metabolic pathways, our primary interest is to increase resistance to cricket pathogens and improve the nutritional content of crickets. Therefore, we examined the transcriptome for sequences and expression levels associated with antibiotic production and hexamerin storage proteins in these initial studies, and identified genes typically associated with RNAi that were included in the transcriptome.

One of the striking findings in the KEGG pathway analysis was a large number of enzymes in antibiotic synthesis pathways. One group of enzymes was similar to prolixicin, which is antibacterial and has an attacin functional domain. We further evaluated the expression of contigs encoding prolixicin and found an increase in expression correlated to early feeding stages. Increased expression of antimicrobial genes in young nymphs may be biologically significant since these young crickets are new to exploring their environment and foraging for food that may contain microbial pathogens. However, expression declined in mature nymphs, but was again increased in adults, more so in females compared to males. One prolixicin contig (cl_100345_1) was expressed at relatively higher levels in all developmental stages. A prolixicin gene was first described in the kissing bug, *Rhodnius prolixus*, a major vector of *Trypansoma cruzi*^42^. The *R. prolixus* prolixicin gene encodes a glycine-rich antibacterial peptide of 11 kDa, and the gene was expressed 200-fold higher with bacterial challenge, with a 500-fold increase in parasite challenged insects. Our results suggest that this antibacterial peptide in the cricket may be important in pathogen protection early in life, and again may be more important in adult females. More research is needed in this area, as there were many genes related to antibiotic production in *A. domesticus*, and these peptides may have other functions in the insect.

We were particularly interested in hexamerins as they are major storage proteins in insects and accumulate to very high levels in larvae^43^. There were 101 contigs annotated as hexamerin in the *A. domesticus* transcriptome, but a genome assembly is needed to provide a better understanding of the exact number of genes encoding hexamerin. However, there was a significant difference (p < 0.05) in the expression of 14 hexamerin contigs in different life stages of *A. domesticus*. As with other gene groups, the expression patterns of hexamerins were grouped to those expressed more in early stages (embryos and hatchlings) and others in later stages (nymphs and adults). Hexamerins also have functions other than storage proteins, as they bind hormones or other small organic molecules, are involved in cross-linking cuticle, as well as protection in humoral immune defense^43^. Hexamerins also may be involved in allergic reactions, as hexamerin 1B was identified as an allergen specific to *G. bimaculatus*^44^. Identifying potential allergens in edible insects is an ongoing effort in the insect food industry^45,46^. Surprisingly, the only hexamerin sequence in the parasitoid wasp *Bracon hebetor* was found in the venom^47^ and also was identified as an allergen in honeybee venom (*Apis mellifera*)^48^. More work is needed to understand hexamerins related to the protein content of crickets, and whether they have a role in allergenicity.

The finding of transcripts from *G. niphandrodes* in this cricket transcriptome assembly suggests that these crickets contained the gregarine parasite. The low coverage of transcripts may explain why the NCBI filter missed the parasite transcripts in the initial assembly submitted to TSA, and during the analysis of data for this study, these contigs were removed and placed in a separate accession. Gregarines are in the phylum Apicomplexa subclass Gregarinasina and are host-specific for invertebrates^49^. The notable exception is the recent inclusion of vertebrate parasites of the genus *Cryptosporidium* in this subclass^50^. Gregarines do not have vertebrate hosts, and the effect of gregarines on invertebrate hosts has been debated. In crickets, the number of spermatophores was negatively correlated to the gregarine load in *G. veletis* and *G. pennsylvanicus*^51^, and thus impacted reproduction. However, our expression data based on contigs from the gregarine *G. niphandrodes* indicated that male adults had a lower load of gregarines than females and nymphs. Since prolixicin has been demonstrated to have increased expression in parasite-challenged insects, the sharp increase in expression of transcripts encoding prolixicin and those from *G. niphandrodes*, both observed in 1 wk nymphs, may be related, but more research is needed to confirm the association.

We found all components known to be necessary for a robust RNAi response in the *A. domesticus* transcriptome assembly. RNAi of the *nubbin* gene in *A. domesticus* demonstrated its role in appendage formation^52^. Injected dsRNA reduces gene expression in other cricket species, as RNAi of a gene encoding a male accessory gland serine protease was used to disrupt the induction of egg-laying in females in an *Allonemobius* spp.^53^. As mentioned previously, RNAi also has been used to evaluate segmentation patterns and leg regeneration in *G. bimaculatus*^15,16^. Genetic engineering of crickets for food production will rely on the alteration of genes for optimization of food content and disease protection, including both RNAi and CRISPR/Cas9 systems. The data in this study provide a first glimpse of information that will be vital for these processes.

## Conclusions

The present study represents the first comprehensive data of transcripts from six developmental stages and male and female adults of *A. domesticus*. We provide examples of data mining prolixicin transcripts for the development of disease-resistant crickets, and hexamerin related transcripts for improved protein content in insects. Sequences associated with RNAi in other insects, as well as those useful for genetic engineering, were identified in the *A. domesticus* transcriptome. These data are critical in the development of genetic resources to improve crickets and other insect species for human food and animal feed production.

## Supplementary information

Supplementary information.

Supplementary information 2.

Supplementary information 3.

Supplementary information 4.

Supplementary information 5.

## Data Availability

All data has been deposited at NCBI as indicated in the Methods. The datasets generated during and/or analyzed during the current study are available from the corresponding author on reasonable request.
